# Radiotherapy Versus Surgery–Which Is Better for Patients With T1-2N0M0 Glottic Laryngeal Squamous Cell Carcinoma? Individualized Survival Prediction Based on Web-Based Nomograms

**DOI:** 10.3389/fonc.2020.01669

**Published:** 2020-08-26

**Authors:** Yajing Du, Shali Shao, Minghe Lv, Yi Zhu, Li Yan, Tiankui Qiao

**Affiliations:** ^1^Center for Tumor Diagnosis and Therapy, Jinshan Hospital, Fudan University, Shanghai, China; ^2^Department of Radiation Oncology, Eye and ENT Hospital, Fudan University, Shanghai, China

**Keywords:** glottic laryngeal squamous cell carcinoma, radiotherapy, surgery, SEER, nomogram

## Abstract

**Background:**

Both radiotherapy and surgery are now recommended for early stage glottic laryngeal squamous cell carcinoma (LSCC), and both have their own advantages in patients with different characteristics. For each patient, it is hard to determine whether radiotherapy or surgery is more appropriate.

**Methods:**

Patients with T1-2N0M0 glottic LSCC who received radiotherapy or surgery in the 2004–2016 SEER database were reviewed, then randomly divided into training and validation cohorts. Propensity score matching was used to eliminate the baseline variations, and competing risk analyses helped to exclude the effects of other causes of death. Based on univariate and multivariate analyses, we built two nomograms to visually predict the survival of each patient with different characteristics who received radiotherapy or surgery, then validated the accuracy in both training and validation cohorts. Using nomogramEx, we quantified the algorithms of the nomograms and put the nomograms on the websites.

**Results:**

A total of 6538 patients in the SEER database were included. We found that therapy (*p* = 0.004), T stage (*p* < 0.001), age (*p* < 0.001), race (*p* < 0.044), grade (*p* = 0.001), and marital status (*p* < 0.001) were independent prognostic factors. Two nomograms were built to calculate the survival for each patient who received radiotherapy (C-index = 0.668 ± 0.050 in the training cohort and 0.578 ± 0.028 in the validation cohort) or underwent surgery (C-index = 0.772 ± 0.045 in the training cohort and 0.658 ± 0.090 in the validation cohort). Calibration plots showed the accuracy of the nomograms. Using the nomograms, we found that 3872 patients (59.22%) had no difference between the two therapies, 706 patients (10.80%) who received radiotherapy had better survival outcomes, and 1960 patients (29.98%) who underwent surgery had better survival outcome.

**Conclusion:**

Nomograms were used to comprehensively calculate independent factors to determine which treatment (radiotherapy or surgery) is better for each patient. A website was used to offer guidance regarding surgery or radiation for patients and physicians.

## Introduction

Laryngeal cancer occurs more frequently in head and neck cancers, and approximately 95% of which are laryngeal squamous cell carcinomas (LSCCs) (1). In China, the incidence of laryngeal cancer is approximately 1.86 per 100,000 annually (2). A total of 23,400 new cases occurred in 2014 (3), most of which were diagnosed in the early stage.

The recommended treatment for early glottic LSCC includes surgery and radiotherapy (4–7). Glottic LSCC is the main site of laryngeal squamous cell carcinomas. In our previous research, we found that the survival of patients with T1a glottic cancer, well-differentiated tumors, who were married, and who received radiotherapy were worse. For patients who had T1b glottic cancer, undifferentiated tumors, and who were unmarried, radiotherapy was not preferable to surgery (8). Individual patients have a complex combination of clinical characteristics, and further exploration of individualized treatment methods for patients with early stage glottic LSCC is warranted to personalize treatment (9–11).

The Surveillance, Epidemiology, and End Results (SEER) program is a source for long-term population-based incidence data. In recent years, nomograms have frequently been used to calculate the proportion of various factors for each patient, and comprehensively consider the impact of multiple factors on survival, which may offer guidance for individual treatment. In this manuscript, we attempted to determine which therapy (radiation or surgery) is a better choice for a patient with T1-2N0M0 glottic LSCC using SEER data and a nomogram.

## Materials and Methods

### Ethics Statement

The Ethics Committees of Jinshan Hospital and the Eye and ENT Hospital of Fudan University exempted the study because no personal information is included in the SEER database.

### Data Selection

**FIGURE 1:**
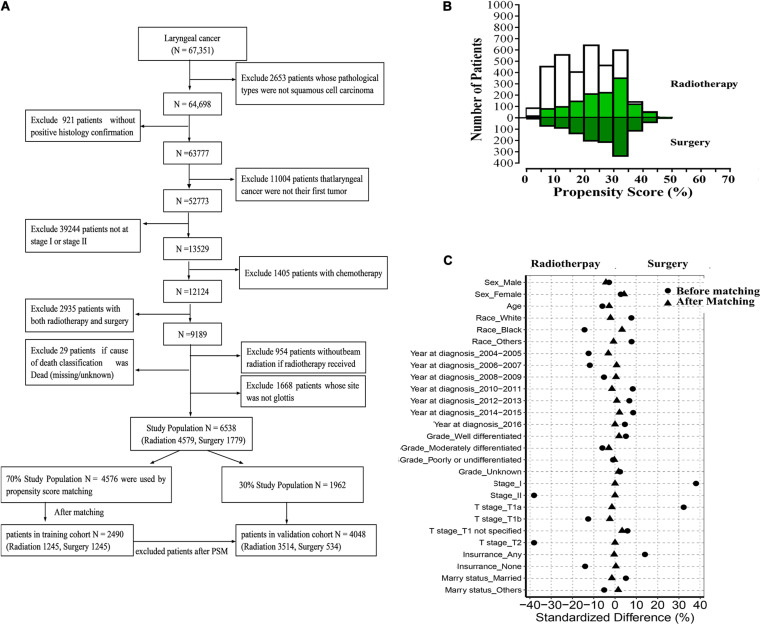
**(A)** Flow diagram of selecting process **(B)** Mirror histogram of propensity scores for patients with radiotherapy and with surgery. Matched patients are presented in green color. **(C)** Standardized differences of baseline variables between patients with radiation and with surgery before and after propensity score matching.

We obtained SEER (Incidence – SEER 18 Regs Custom Data with additional treatment fields, November 2018 Sub, 1975 – 2016 varying) data via the SEER^∗^Stat software (version 8.3.6)^1^. The selection process to acquire data from the database is shown in Figure 1A. In brief, we selected patients who had early stage glottic LSCC and underwent only radiotherapy or surgery. The old version to the 8th AJCC TMN staging system was converted manually.

Characteristics, including race, age, gender, grade, TMN stage, T stage, marital status, and insurance, were included in the analyses.

### Study Design and Statistical Analysis

Statistical analyses were performed using IBM SPSS Statistics 25.0 (IBM, Inc., Armonk, NY, United States) and R version 3.6.1 (R Foundation for Statistical Computing, Vienna, Austria). A two-tailed *p*-value < 0.05 was considered statistically significant.

Patients were divided into two groups (radiotherapy and surgery) and were randomly divided into training and validation cohorts. A propensity score matching system was used to eliminate patient selection bias in the training cohort. In the current study, covariates that may affect the choice of grouped patients were matched as follows: age (≤50, 51–60, 61–70, 71–80, and ≥81 years); race; gender; grade; TMN stage; T stage; and insurance and marital status. Patients who were excluded after propensity score matching were moved to the validation cohort to improve the accuracy of the validation process.

Propensity scores for the training cohort were generated using the “MatchIt” package. The baseline characteristics between the surgery and radiotherapy groups before and after matching were compared using χ2 and Wilcoxon tests (12, 13). All of the patients except the propensity scoring matches were divided into the validation cohort.

The Kaplan-Meier method was used to estimate survival rates. Survival curves were compared using a log-rank test. Competing risk analyses were performed as previously reported (14) because other causes of death were competing outcomes for cancer-specific deaths. A Cox proportional hazards model was used to perform univariate and multivariate analyses.

Two nomograms, as several studies have reported (15, 16), were built on the basis of the results of multivariate analysis. The performance of nomograms was evaluated by the concordance index (C-index). The nomograms were also assessed by comparing the actual probability with the predicted probability and were further validated by comparing the predicted probability in the validation cohort with the observed survival. The “nomogramEx” package was used to extract the polynomial equations to calculate the points of each variable for every patient, and the survival probability corresponding to the total points. Using nomogramEx, the cancer-specific survival rates for each patient who was treated with radiation or underwent surgery at 3 and 5 years were calculated.

## Results

### Patient Characteristics

As shown in Figure 1A, 6538 patients with glottic LSCC (4759 patients treated with radiation and 1779 patients who underwent surgery) were included in our study. A total of 4576 patients were randomly divided into the training cohort. Patients who were excluded after propensity score matching comprised the validation cohort. The baseline characteristics of all participants in the training cohort are summarized in Table 1 and Supplementary Table S1. Compared to the patients who underwent surgery, the patients who underwent radiotherapy were characterized as follows: older (*p* = 0.071); worse tumor differentiation (*p* = 0.259); higher T (*p* < 0.001) and TMN stage (*p* < 0.001); less likely to be white (*p* < 0.001); less likely to have insurance (*p* < 0.001), and less likely to be married (*p* = 0.134).

**TABLE 1:** Patient characteristics according to the therapy status before and after propensity score matching.

Characteristics	Before matching				After matching			
	Radiation	Surgery	SD (%)	*p*-value	Radiation	Surgery	SD (%)	*p*-value
**Total number**	3331	1245			1245	1245		
**Sex**				0.431				0.315
Male	2954	1093	–2.766		1110	1093	–4.277	
Female	377	152	2.766		135	152	4.277	
**Age**				0.111				0.966
∼50	268	127	7.490		123	127	1.069	
51∼60	821	309	0.399		322	309	–2.401	
61∼70	1091	378	–5.148		375	378	0.525	
71∼80	802	289	–2.034		279	289	1.914	
81∼	349	142	2.974		146	142	–1.005	
**Race**				<0.001				0.709
White	2785	1075	7.666		1084	1075	–2.129	
Black	421	103	–14.304		92	103	3.289	
Others	125	67	7.808		69	67	–0.707	
Year at diagnosis*				<0.001				0.987
**Grade**				0.253				0.896
Well differentiated	728	299	5.141		289	299	1.891	
Moderately differentiated	1516	530	–5.928		548	530	–2.918	
Poorly or undifferentiated	254	92	–0.895		93	92	–0.306	
Unknown	833	324	2.332		315	324	1.655	
**Stage**				<0.001				1
I	2451	1099	38.066		1099	1099	0.000	
II	880	146	–38.066		146	146	0.000	
**T stage**				<0.001				0.815
T1a	1439	736	32.252		745	736	–1.473	
T1b	355	88	–12.654		96	88	–2.456	
T1 not specified	657	275	5.817		258	275	3.330	
T2	880	146	–38.066		146	146	0.000	
**Insurance status at diagnosis**				<0.001				0.961
Any	2442	987	14.063		989	987	–0.397	
None or unknown	889	258	–14.063		256	258	0.397	
**Marital status at diagnosis**				0.134				0.741
Married	1969	767	5.102		776	767	–1.489	
Others	1362	478	–5.102		469	478	1.489	

Asterisk “*” refers to the year the patient was diagnosed with the disease.

### Propensity Score Matching Analyses

After matching, 1245 pairs of patients were selected; one-half received radiotherapy, and the other half underwent surgery. As shown in Table 1 and Supplementary Table S1, there were no significant differences between the radiotherapy and surgery groups after matching. The *p*-values for variables, including age, race, year of diagnosis, grade, stage, T stage, insurance, and marital status, had been greatly improved. The absolute values of the standardized differences (SD) after matching were all <10%, suggesting that the baseline characteristics were well-balanced. The matched groups had similar propensity score distributions, and the mirror histograms of propensity scores for patients are shown in Figures 1B,C.

### Survival Analyses

**FIGURE 2:**
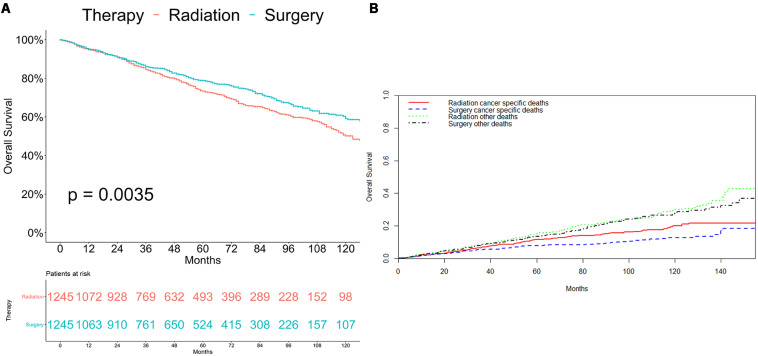
Survival analyses for patients with radiotherapy and with surgery **(A)** Kaplan-Meier method. **(B)** Competing risk analysis.

As shown in Figure 2, patients who received radiation therapy had significantly worse overall survival outcomes compared with patients who underwent surgery (*p* = 0.0035; Figure 2A). Competing risk analysis also illustrated that the patients who received radiation had a higher risk of cancer-specific mortality (*p* = 0.003), while there was no apparent difference in the probabilities of other causes of death (*p* = 0.351; Figure 2B).

We further analyzed the connection between cancer-specific survival and variations. Multicollinearity was detected to test the independence of the variables included in the regression model, and variance inflation factors (VIF) of all variables were far less than ten indicates there was no multicollinearity problem. As Table 2 and Supplementary Table S2 shows, univariate analyses demonstrated that therapy, age, race, grade, stage, T stage, and marital status were significant predictors of glottic LSCC-specific survival. Gender, year of diagnosis, and insurance status had no significant differences between the two groups. Based on multivariate analysis for patients with glottic LSCC, therapy, race, age, grade, T stage, and marital status were independent prognostic predictors and stage, which were not independent of T stage and not included in the multivariate analysis.

**TABLE 2:** Results of univariate and multivariate analyses of cancer-specific survival after matching.

Characteristics	Univariate analyses				Multivariate analysis		
	HR	95% CI	*p-*value	VIF	HR	95% CI	*p-*value
**Therapy**			0.004	1.001			0.002
Radiation	Reference				Reference		
Surgery	0.795	0.680–0.928	0.004		0.783	0.670–0.915	0.002
**Sex**			0.340	1.023	Not included		
Male	Reference						
Female	1.122	0.886–1.422	0.340				
**Age**			<0.001	1.005			<0.001
<50	Reference				Reference		
51∼60	2.803	1.726–4.551	<0.001		2.756	1.696–4.480	<0.001
61∼70	3.541	2.205–5.688	<0.001		3.679	2.289–5.913	<0.001
71∼80	6.119	3.820–9.802	<0.001		6.296	3.928–10.090	<0.001
81∼	11.341	6.998-18.379	<0.001		11.318	6.980–18.350	<0.001
**Race**			0.017	1.009			0.044
White	Reference	–			Reference		
Black	1.026	0.774–1.359	0.859		0.933	0.700–1.244	0.638
Others	0.525	0.336–0.820	0.005		0.568	0.363–0.889	0.013
Year at diagnosis*			0.809	1.564	Not included		
**Grade**			0.009	1.005			0.001
Well differentiated	Reference				Reference		
Moderately differentiated	1.117	0.911–1.368	0.287		1.126	0.917–1.382	0.257
Poorly or undifferentiated	1.655	1.233–2.221	0.001		1.794	1.334–2.413	0.000
Unknown	1.144	0.913–1.432	0.242		1.184	0.943–1.485	0.145
**Stage**			<0.001	Not included	Not included		
I	Reference						
II	1.476	1.203–1.810	<0.001				
**T stage**			<0.001	1.013			<0.001
T1a	Reference				Reference		
T1b	1.227	0.907–1.660	0.184		1.279	0.943–1.734	0.114
T1 not specified	1.200	0.988–1.458	0.066		1.180	0.971–1.434	0.097
T2	1.573	1.271–1.949	<0.001		1.641	1.323–2.035	<0.001
**Insurance status**			0.120	1.557	Not included		
Any	Reference						
None or unknown	1.149	0.965–1.367	0.120				
**Marital status**			<0.001	1.024			<0.001
Married	Reference				Reference		
Others	1.509	1.292–1.763	<0.001		0.676	0.577–0.792	<0.001

Asterisk “*” refers to the year the patient was diagnosed with the disease.

To better compare the difference of treatment on cancer-specific survival in glottic LSCC patients, we stratified the patients after matching by variables in univariate analysis. As is shown in Supplementary Figures S1–S8, glottic LSCC patients with a worse cancer-specific survival had the following characteristics: not black; male; insurance; T1a stage; well-differentiated tumor; married, 51–60 or 71–80 years of age. For glottic LSCC patients with other characteristics, radiotherapy and surgery had equivalent efficacy.

### Construction and Validation of the Nomogram

**FIGURE 3:**
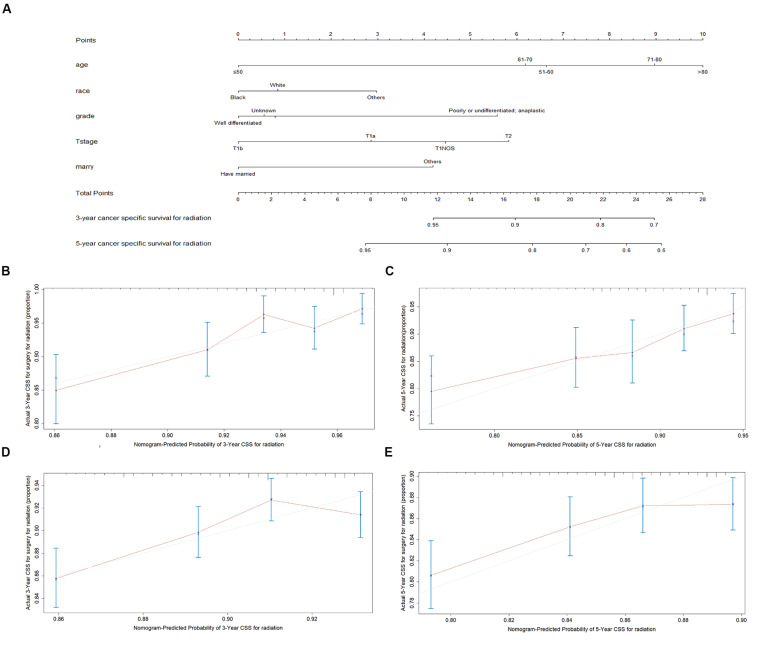
Nomogram analyses for patients with radiotherapy **(A)** A nomogram for prediction of 3- and 5-year CSS rates of patients **(B)** Calibration curve of the nomogram predicting 3-year CSS rates in training cohort. **(C)** Calibration curve of the nomogram predicting 5-year CSS rates in training cohort. **(D)** Calibration curve of the nomogram predicting 3-year CSS rates in validation cohort **(E)** Calibration curve of the nomogram predicting 3-year CSS rates in validation cohort.

**FIGURE 4:**
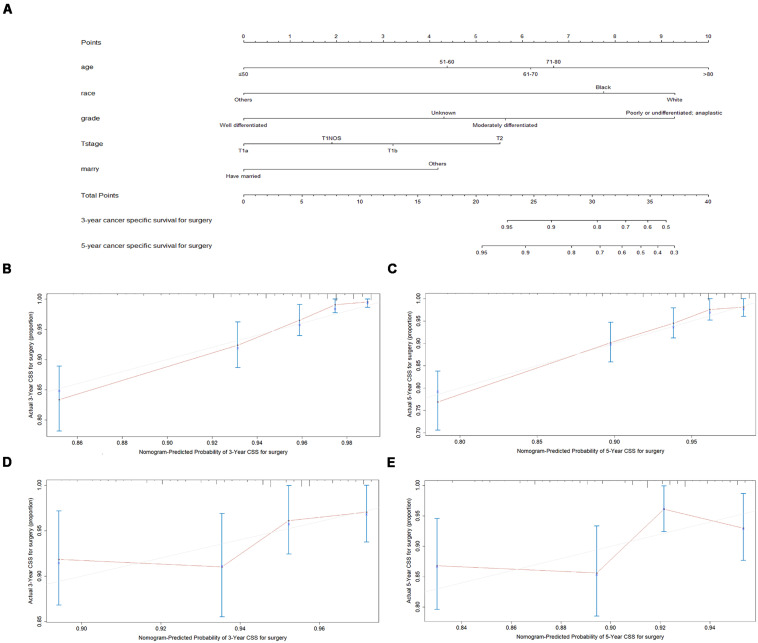
Nomogram analyses for patients with surgery **(A)** A nomogram for prediction of 3- and 5-year CSS rates of patients **(B)** Calibration curve of the nomogram predicting 3-year CSS rates in training cohort. **(C)** Calibration curve of the nomogram predicting 5-year CSS rates in training cohort. **(D)** Calibration curve of the nomogram predicting 3-year CSS rates in validation cohort **(E)** Calibration curve of the nomogram predicting 3-year CSS rates in validation cohort.

The final multivariate model uncovered six independent variables, including therapy, age, race, grade, T stage, and marital status. As shown in Figures 3A, 4A, the nomograms were developed based on the variables.

A total of 4576 patients were randomly divided into the training cohort and patients excluded after propensity score matching comprised the validation cohort. As shown in Figures 3B,C, 4B,C, the calibration plots are based on internal validation of the training cohort. The C-index for the prediction of cancer-specific survival in glottic LSCC patients who underwent surgery and received radiotherapy was 0.772 ± 0.045 and 0.668 ± 0.050, respectively. In addition, external validation of the nomograms was performed in the validation cohort. The C-index for surgery (0.658 ± 0.090) and radiotherapy (0.578 ± 0.028) was calculated based on the calibration plots shown in Figures 3D,E, 4D,E. The C-index for internal and external validation indicated that the nomograms have a good fit with the actual observations. Therefore, the 3- and 5-year cancer-specific survival rates predicted by the nomograms were reliable.

### Nomogram Analyses and Website Application

We used the nomogramEx function in R software to calculate the specific algorithms for the above two nomograms and uploaded the algorithms to our website^2^. Patients and physicians can enter age, grade, marital status, and other personal details on our website, which automatically calculates the predicted 3- and 5-year survival rates of patients who received radiotherapy or underwent surgery. The patients and physicians can compare outcomes of radiation and surgery to determine which is the better treatment option. For example, an 82-year-old Chinese patient with a stage T1a moderately differentiated glottic LSCC who is divorced can use our website to find out that the 3- and 5-year survival rates for surgery are 98.16 and 94.78%, respectively, while the corresponding rates for radiotherapy are 78.40 and 68.45%, respectively. At this time, surgery may be a better choice for this patient.

**FIGURE 5:**
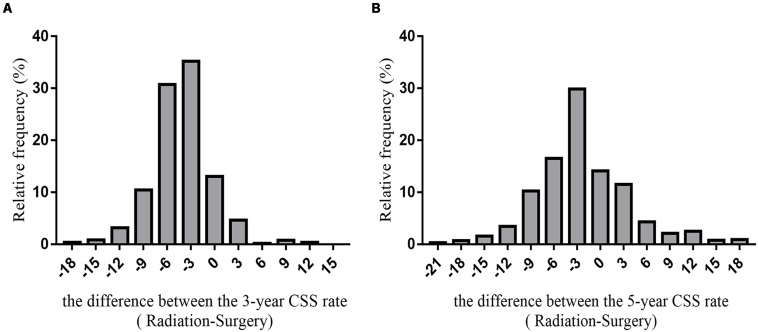
The histogram of the difference between CSS rates (radiotherapy-surgery) **(A)** the difference between 3-year CSS rates **(B)** the difference between 5-year CSS rates.

We predicted the prognosis of patients with early glottic LSCC who underwent surgery and radiotherapy. Greater than 6000 patients in the SEER databases were included in this study, and we listed the results as a histogram in Figure 5.

The average difference between the 3-year cancer-specific survival rates assuming all patients received radiotherapy or underwent surgery was −4.29 ± 3.94%. The difference between the 5-year cancer-specific survival rates assuming all patients received radiotherapy or underwent surgery was −2.04 ± 6.74%.

Based on the SEER database, the 5-year survival rate for surgery for 1960 patients (29.98%) was >5% higher than the 5-year survival rate for radiotherapy. The 5-year survival rate for 706 patients undergoing surgery was <5% than the 5-year survival rate for patients who received radiotherapy. The difference in the 5-year survival rate between 3872 patients (59.22%) who underwent surgery or received radiotherapy was between −5 and 5%; thus, this group of patients may choose surgery or radiotherapy.

## Discussion

In this study, we compared the cancer-specific survival of 6538 patients with early stage glottic LSCC, who received radiotherapy or underwent surgery. We built and validated two web-based nomograms to predict the cancer-specific survival for each patient who received radiotherapy or underwent surgery. Patients can input relevant information, such as age, grade, T stage, and marital status, on our website and estimate which treatment is superior. Our findings will be of great benefit to help patients and physicians to make treatment decisions.

We showed that radiotherapy and surgery each have their own advantages in the glottic LSCC population (17). Of the 6538 patients from the SEER database, surgery was superior in 29.98%, radiotherapy was superior in 10.80%, and operation and radiotherapy had similar efficacy in 59.22%. Patients and physicians can use our web-based nomograms to predict which therapy is more appropriate for them.

A nomogram is a simple graphical representation of a statistical prediction model that is frequently applied to patients and has gained popularity among oncologists and patients participating in clinical trials (18–23). In the current study, we used two nomograms to visually and individually predict the survival of glottic LSCC patients with different clinicopathologic characteristics to help them choose a superior treatment modality. We believe that our nomogram-based method can be used to compare the outcomes of several therapies and can play an increasingly significant role in future clinical analyses.

Inverso et al. (24) reported that marital status has a positive effect on metastatic laryngeal cancer. Common symptoms of laryngeal cancer included hoarseness, otalgia, dysphagia, and voice changes (25). Such symptoms can attract the early attention of partners or spouses, who may urge patients to receive timely diagnosis and treatment. Investigators have reported that patients who have fee-for-service insurance are more likely to undergo cancer screening tests that may affect stage at the time of diagnosis (26). Our analysis showed that insurance was not an independent factor in patients with early stage laryngeal cancer. The relationship between insurance and survival outcome needs further exploration.

Our study had several limitations. First, the information in the SEER database is not detailed, such as radiation technology, radiation dose, and surgery regimen. We were not able to calculate the influence of these factors. Second, owing to the absence of life quality data in the SEER database, we only focused on survival outcome rather than assess functional outcomes, while a trial revealed that patients with radiotherapy had less hoarseness-related inconvenience (27, 28). Du et al. (2) reported that surgery had preferable fundamental frequency values over radiotherapy in T1aN0M0 glottic carcinoma (29). With the development of surgery in recent years, the effect of surgery on pronunciation and other functions has greatly improved (7, 30–34). Third, the results might not be applicable to other populations because patients included in this research were from the United States. The last, we only analyzed the clinical characteristics but didn’t include several molecular factors such as HPV, or TP53 mutations since such information was not included in the SEER database. The last, but most important limitation was that it should be noted that our results may not be a reference before prospective trials are conducted because the study was retrospective.

## Conclusion

In our study, we analyzed the independent prognostics factors for early stage glottic LSCC patients and built nomograms to comprehensively calculate independent factors and help determine which treatment, radiotherapy, or surgery, is better for each patient. A website is also available to offer guidance about surgery or radiation for patients and physicians.

## Data Availability Statement

All datasets generated for this study are included in the article/Supplementary Material.

## Ethics Statement

The Ethics Committees of Jinshan Hospital and the Eye and ENT Hospital of Fudan University exempted the study because no personal information is included in the SEER database.

## Author Contributions

YD, SS, and ML: conception and design. YD, LY, and YZ: administrative support. YD, TQ, and SS: provision of study materials or patients. YD and ML: collection and assembly of data. LY, YZ, and TQ: data analysis and interpretation. All authors: manuscript writing and approval of final manuscript.

## Conflict of Interest

The authors declare that the research was conducted in the absence of any commercial or financial relationships that could be construed as a potential conflict of interest.

## Footnotes

1 http://seer.cancer.gov/seerstat/

2 www.datapredictionzc.com/nomogramforLSCC

## Supplementary Material

FIGURE S1 Survival analyses for patients with radiotherapy and with surgery stratified by age after matching **(A)** ≤50 years of age. **(B)** 51–60 years of age. **(C)** 61–70 years of age. **(D)** 71–80 years of age. **(E)** >80 years of age.

FIGURE S2 Survival analyses for patients with radiotherapy and with surgery stratified by race. **(A)** White. **(B)** Black. **(C)** Other race.

FIGURE S3 Survival analyses for patients with radiotherapy and with surgery stratified by sex. **(A)** Male. **(B)** Female.

FIGURE S4 Survival analyses for patients with radiotherapy and with surgery stratified by differentiation after matching **(A)** Well differentiated. **(B)** Moderately differentiated. **(C)** Poorly or undifferentiated, anaplastic. **(D)** Differentiation unknown.

FIGURE S5 Survival analyses for patients with radiotherapy and with surgery stratified by stage. **(A)** Stage I. **(B)** Stage II.

FIGURE S6 Survival analyses for patients with radiotherapy and with surgery stratified by T stage after matching **(A)** T1a. **(B)** T1b. **(C)** T1NOS. **(D)** T2.

FIGURE S7 Survival analyses for patients with radiotherapy and with surgery stratified by insurance. **(A)** Patient who had insurance. **(B)** Patients at other insurance status.

FIGURE S8 Survival analyses for patients with radiotherapy and with surgery stratified by marital status. **(A)** Married patient. **(B)** Patients at other marital status.

TABLE S1 Characteristics of year at diagnosis of patients according to the therapy status before and after propensity score matching.

TABLE S2 Results of year at diagnosis in univariate and multivariate analyses of cancer-specific survival after matching.
