# Supplementary figures and images for: Radiotherapy Versus Surgery–Which Is Better for Patients With T1-2N0M0 Glottic Laryngeal Squamous Cell Carcinoma? Individualized Survival Prediction Based on Web-Based Nomograms
Source: Front Oncol. 2020 Aug 26;10:1669. doi: 10.3389/fonc.2020.01669 (PMC7507900; doi:10.3389/fonc.2020.01669)

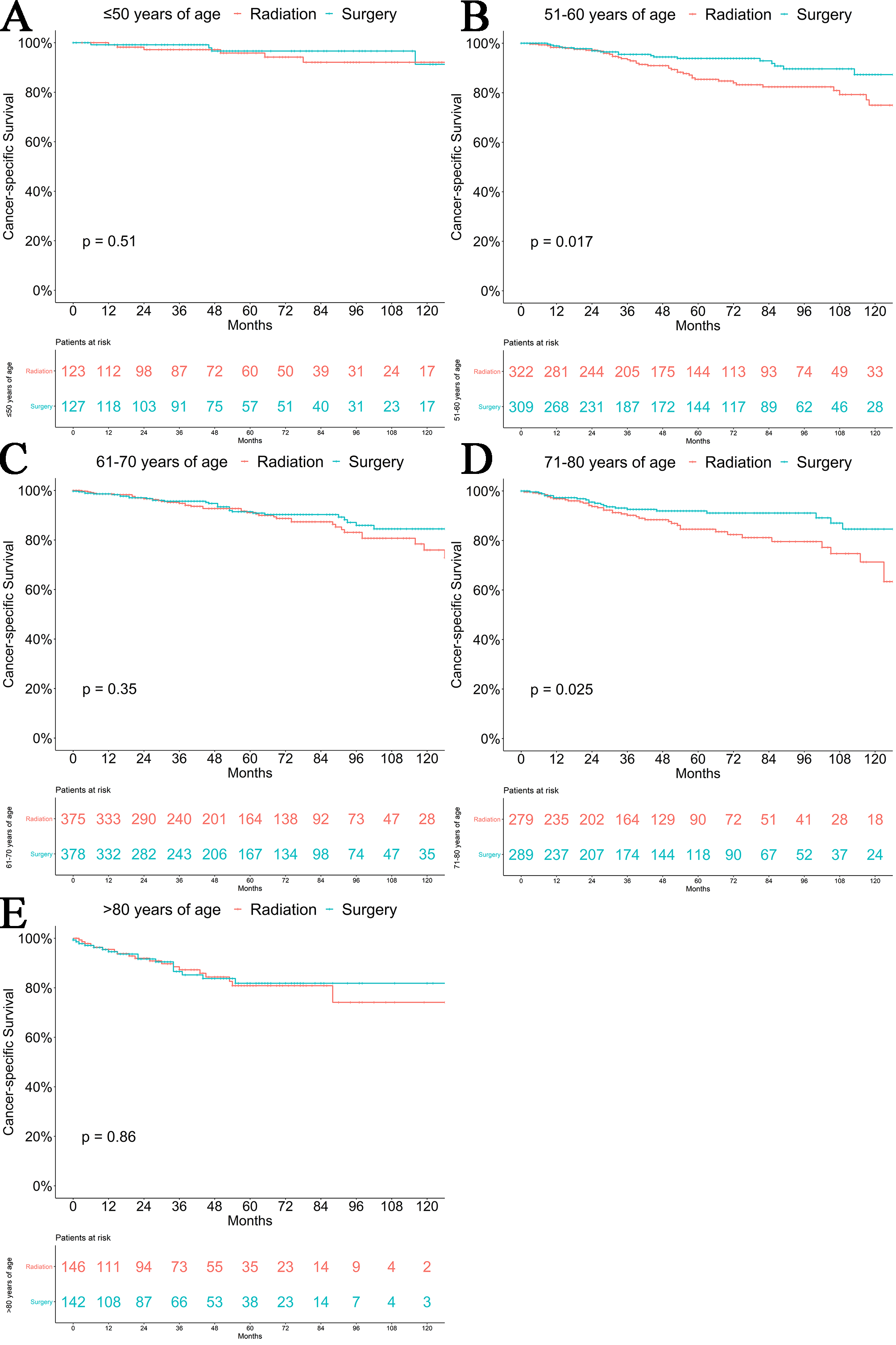

Supplement: FIGURE S1 — Survival analyses for patients with radiotherapy and with surgery stratified by age after matching (A) ≤50 years of age. (B) 51–60 years of age. (C) 61–70 years of age. (D) 71–80 years of age. (E) >80 years of age. [file Image_1.TIF]

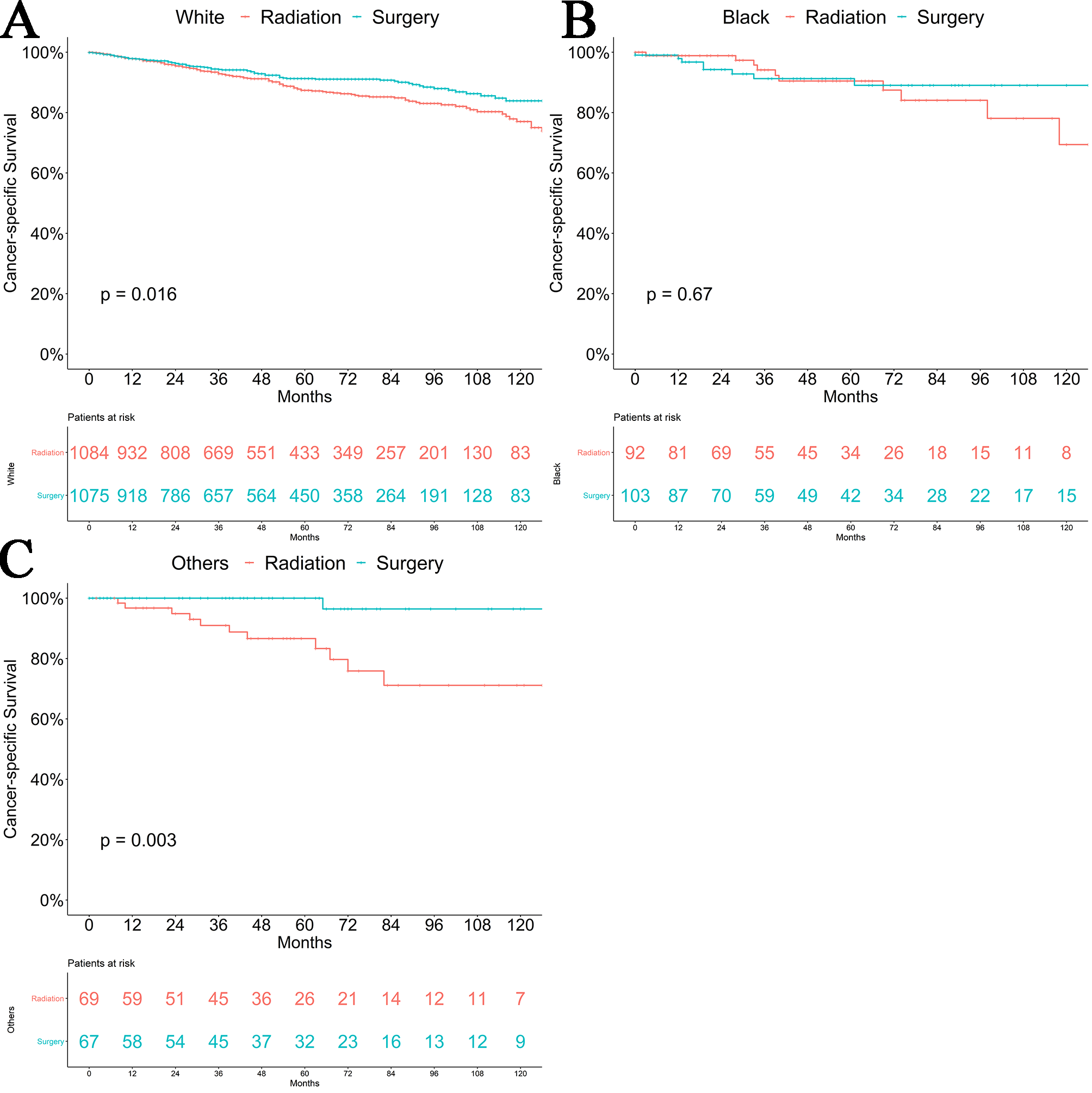

Supplement: FIGURE S2 — Survival analyses for patients with radiotherapy and with surgery stratified by race. (A) White. (B) Black. (C) Other race. [file Image_2.TIF]

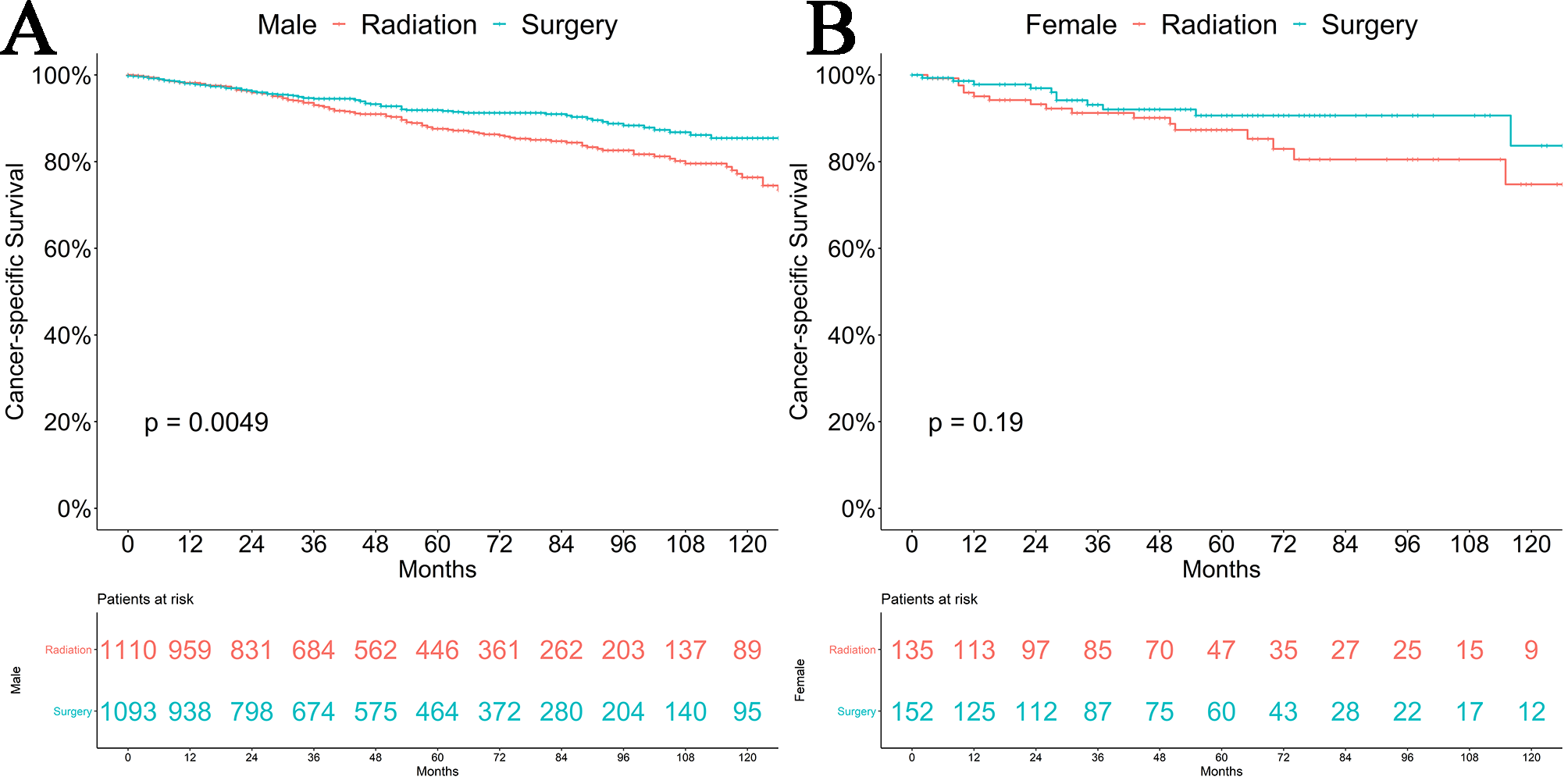

Supplement: FIGURE S3 — Survival analyses for patients with radiotherapy and with surgery stratified by sex. (A) Male. (B) Female. [file Image_3.TIF]

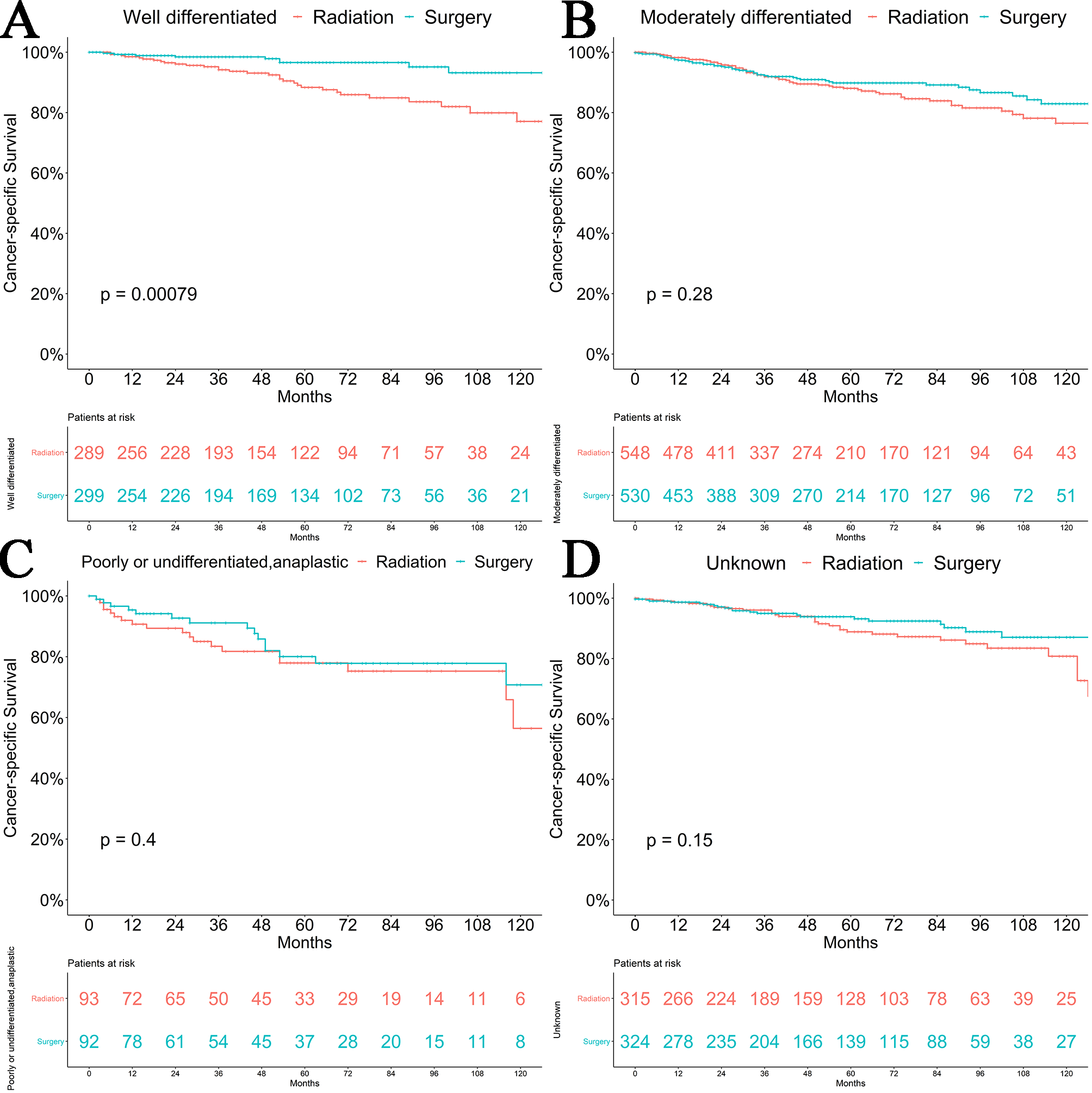

Supplement: FIGURE S4 — Survival analyses for patients with radiotherapy and with surgery stratified by differentiation after matching (A) Well differentiated. (B) Moderately differentiated. (C) Poorly or undifferentiated, anaplastic. (D) Differentiation unknown. [file Image_4.TIF]

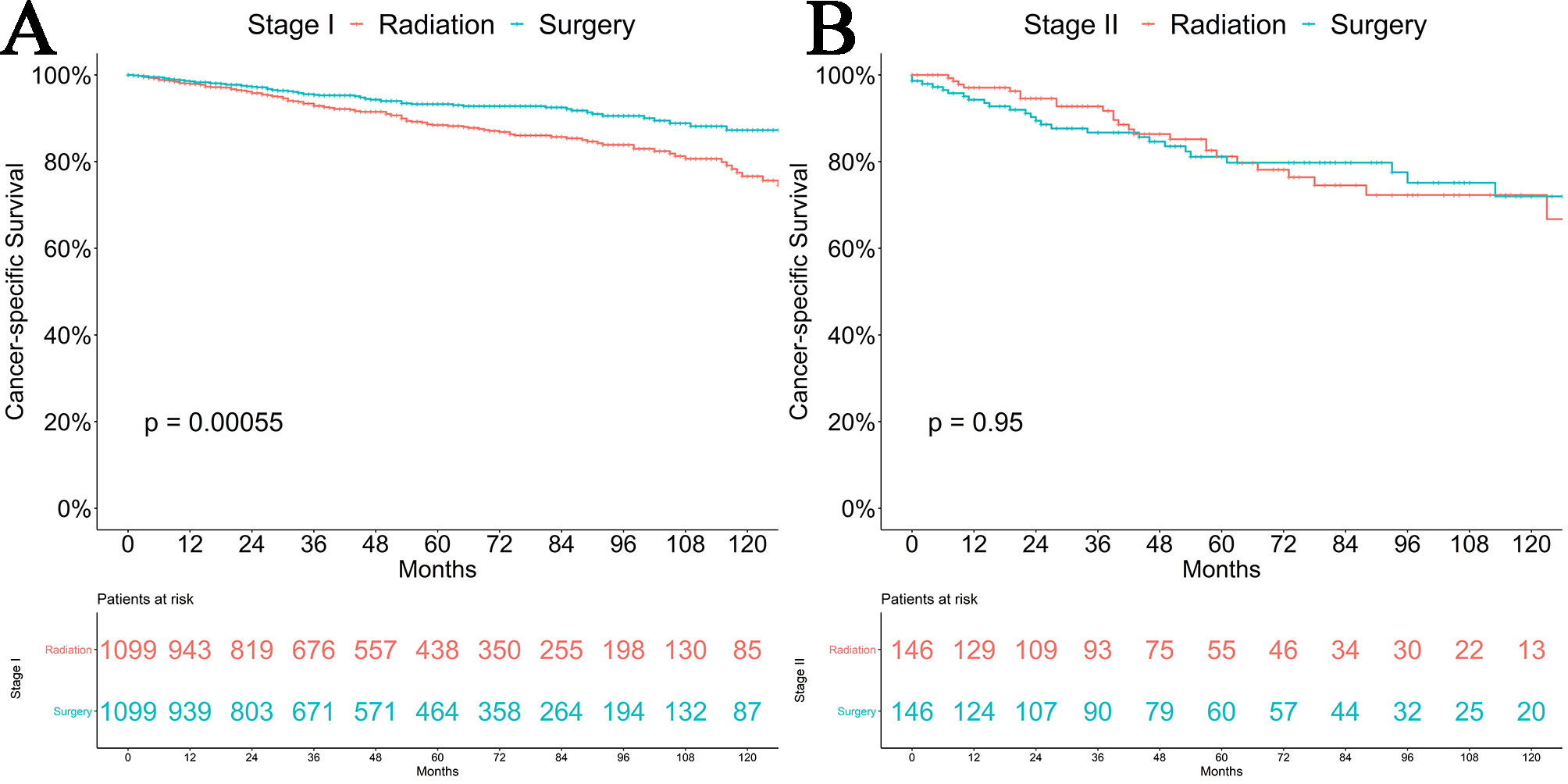

Supplement: FIGURE S5 — Survival analyses for patients with radiotherapy and with surgery stratified by stage. (A) Stage I. (B) Stage II. [file Image_5.TIF]

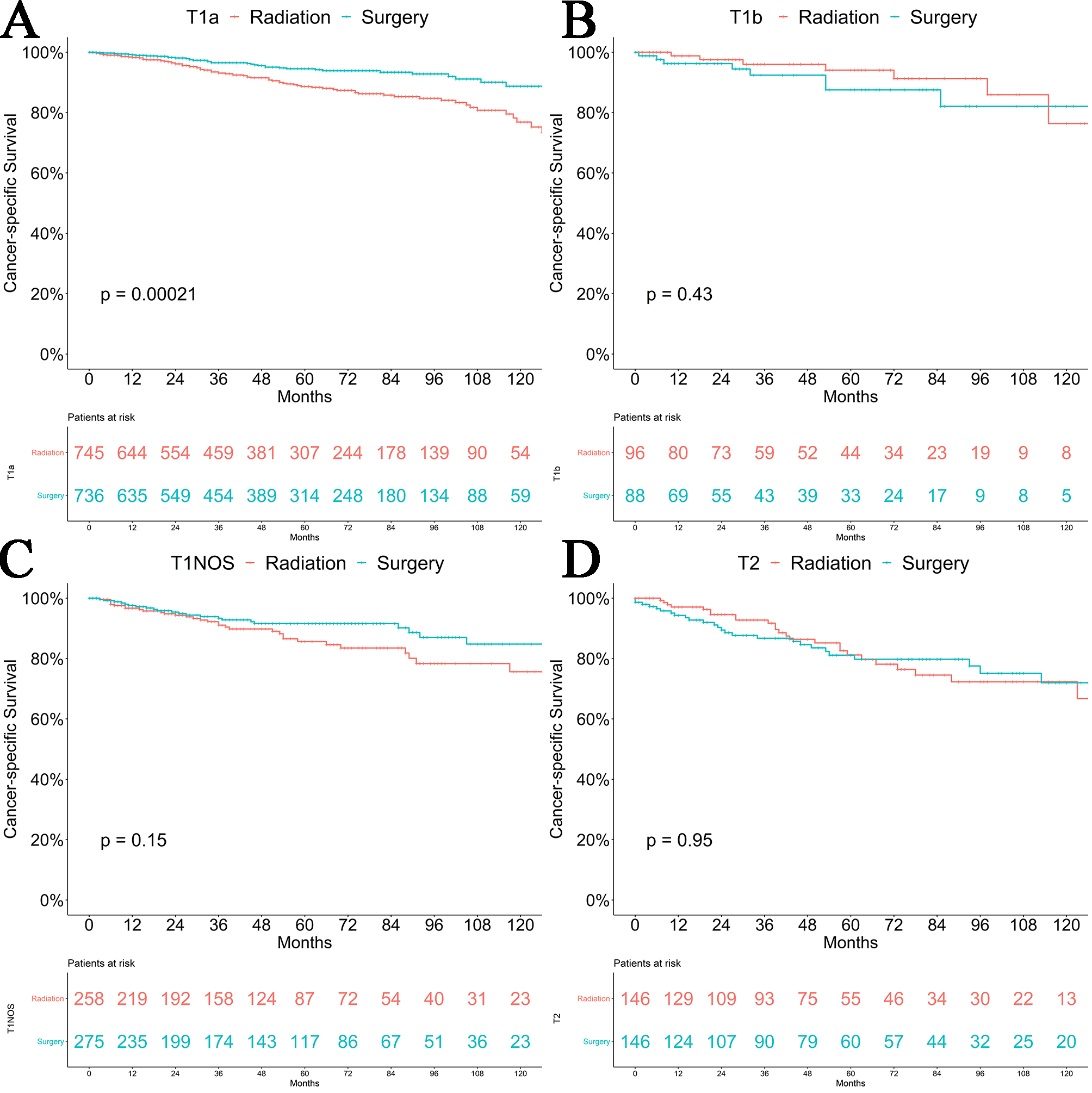

Supplement: FIGURE S6 — Survival analyses for patients with radiotherapy and with surgery stratified by T stage after matching (A) T1a. (B) T1b. (C) T1NOS. (D) T2. [file Image_6.TIF]

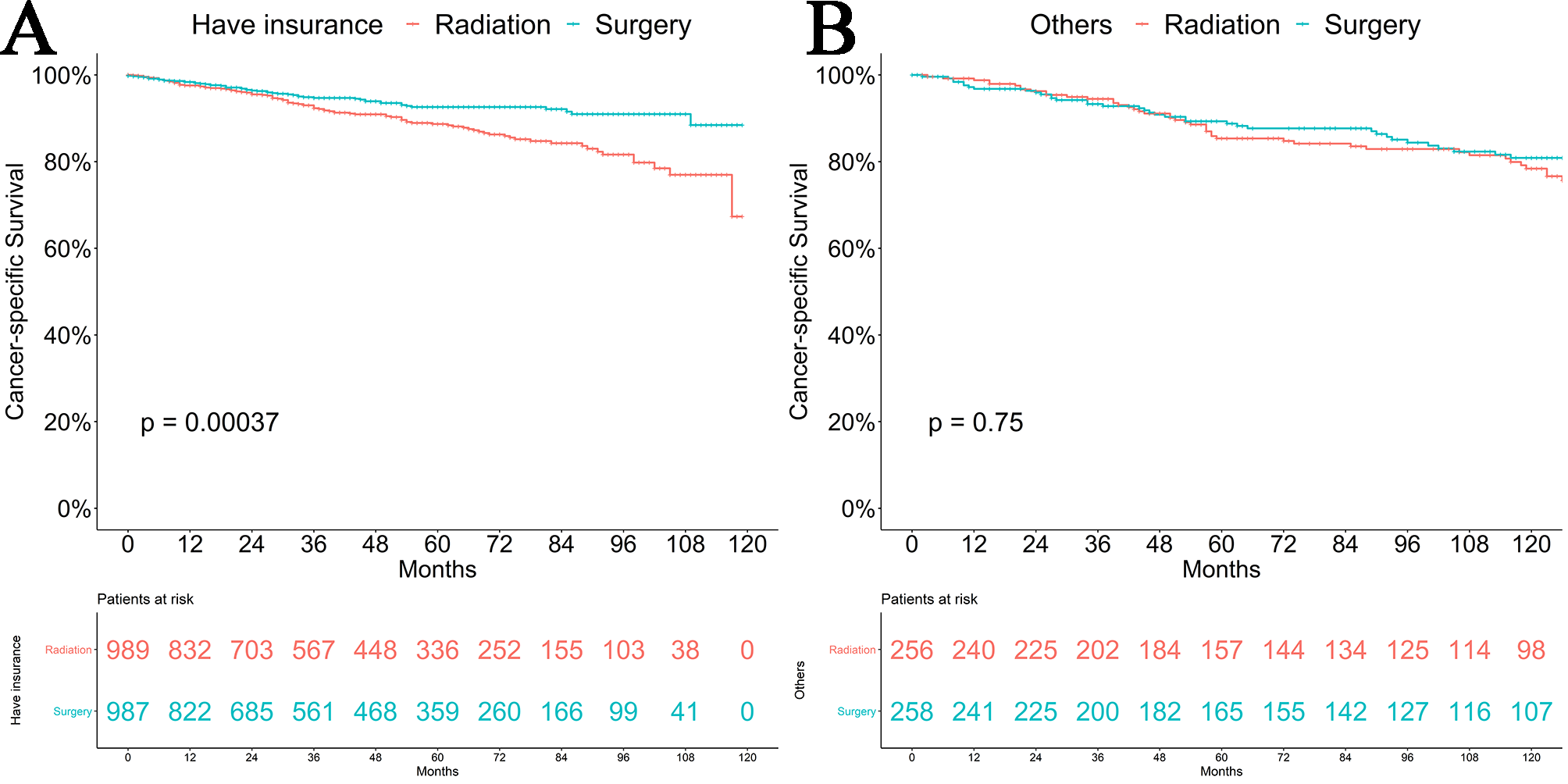

Supplement: FIGURE S7 — Survival analyses for patients with radiotherapy and with surgery stratified by insurance. (A) Patient who had insurance. (B) Patients at other insurance status. [file Image_7.TIF]

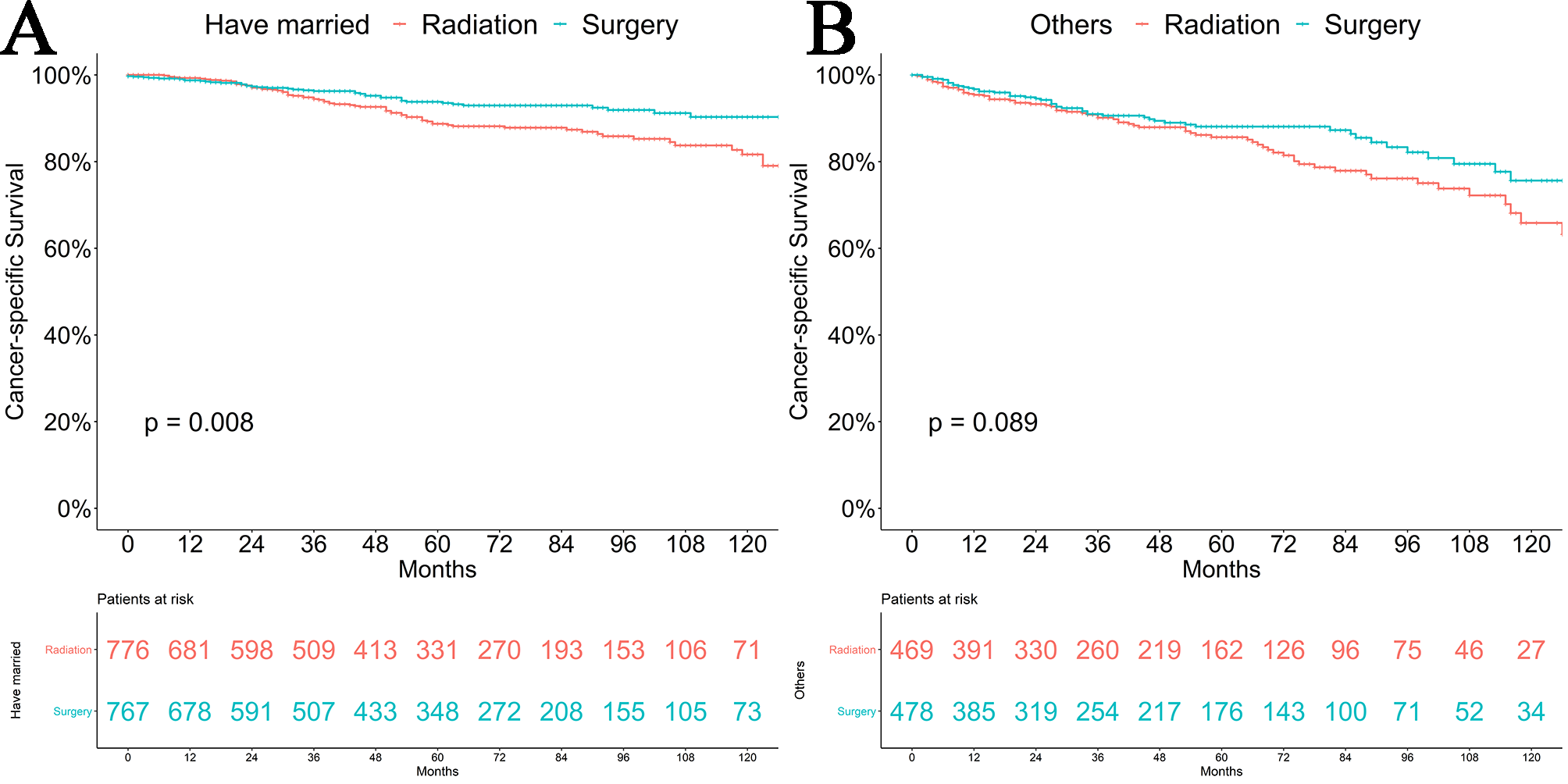

Supplement: FIGURE S8 — Survival analyses for patients with radiotherapy and with surgery stratified by marital status. (A) Married patient. (B) Patients at other marital status. [file Image_8.TIF]
