# Supplementary material for: Radiotherapy Versus Surgery–Which Is Better for Patients With T1-2N0M0 Glottic Laryngeal Squamous Cell Carcinoma? Individualized Survival Prediction Based on Web-Based Nomograms
Source: Front Oncol. 2020 Aug 26;10:1669. doi: 10.3389/fonc.2020.01669 (PMC7507900; doi:10.3389/fonc.2020.01669)
Supplement: TABLE S1 — Characteristics of year at diagnosis of patients according to the therapy status before and after propensity score matching. [file Table_1.docx]

**Table S1** Characteristics of year at diagnosis of patients according to the therapy status before and after propensity score matching.

| **Characteristics** | **Before Matching** | | | |  | | **After Matching** | | | |
| --- | --- | --- | --- | --- | --- | --- | --- | --- | --- | --- |
|  | **Radiation** | **Surgery** | **SD(%)** | ***p* value** | | **Radiation** | | **Surgery** | **SD(%)** | ***p* value** |
| **Total number** | 3331 | 1245 |  |  | | 1245 | | 1245 |  |  |
| **Year at diagnosis*** |  |  |  | <0.001 | |  | |  |  | 0.987 |
| 2004-2005 | 463 | 123 | -12.445 |  | | 135 | | 123 | -3.163 |  |
| 2006-2007 | 551 | 154 | -11.885 |  | | 151 | | 154 | 0.735 |  |
| 2008-2009 | 541 | 179 | -5.178 |  | | 177 | | 179 | 0.459 |  |
| 2010-2011 | 504 | 227 | 8.329 |  | | 234 | | 227 | -1.448 |  |
| 2012-2013 | 513 | 223 | 6.743 |  | | 219 | | 223 | 0.841 |  |
| 2014-2015 | 507 | 229 | 8.493 |  | | 219 | | 229 | 2.091 |  |
| 2016 | 252 | 110 | 4.630 |  | | 110 | | 110 | 0.088 |  |
