# Supplementary material for: Radiotherapy Versus Surgery–Which Is Better for Patients With T1-2N0M0 Glottic Laryngeal Squamous Cell Carcinoma? Individualized Survival Prediction Based on Web-Based Nomograms
Source: Front Oncol. 2020 Aug 26;10:1669. doi: 10.3389/fonc.2020.01669 (PMC7507900; doi:10.3389/fonc.2020.01669)
Supplement: TABLE S2 — Results of year at diagnosis in univariate and multivariate analyses of cancer-specific survival after matching. [file Table_2.docx]

Table S2 Results of year at diagnosis in univariate and multivariate analyses of cancer-specific survival after matching.

| **Characteristics** | **Univariate analyses** | | | **Multivariate analysis** | | |
| --- | --- | --- | --- | --- | --- | --- |
|  | **HR** | **95%CI** | ***p* value** | **HR** | **95%CI** | ***p* value** |
| **Year at diagnosis** |  |  | 0.809 | Not included |  |  |
| 2004-2005 | Reference |  |  |  |  |  |
| 2006-2007 | 0.946 | 0.742-1.206 | 0.655 |  |  |  |
| 2008-2009 | 0.874 | 0.676-1.130 | 0.305 |  |  |  |
| 2010-2011 | 0.869 | 0.668-1.129 | 0.292 |  |  |  |
| 2012-2013 | 0.852 | 0.631-1.150 | 0.296 |  |  |  |
| 2014-2015 | 0.738 | 0.493-1.105 | 0.140 |  |  |  |
| 2016 | 0.770 | 0.277-2.139 | 0.617 |  |  |  |
